# The Quest for Targets Executing MYC-Dependent Cell Transformation

**DOI:** 10.3389/fonc.2016.00132

**Published:** 2016-06-02

**Authors:** Markus Hartl

**Affiliations:** ^1^Institute of Biochemistry and Center of Molecular Biosciences (CMBI), University of Innsbruck, Innsbruck, Austria

**Keywords:** signal transduction, transcription, genetic, oncogenes, tumor suppressor, carcinogenesis

## Abstract

MYC represents a transcription factor with oncogenic potential converting multiple cellular signals into a broad transcriptional response, thereby controlling the expression of numerous protein-coding and non-coding RNAs important for cell proliferation, metabolism, differentiation, and apoptosis. Constitutive activation of MYC leads to neoplastic cell transformation, and deregulated *MYC* alleles are frequently observed in many human cancer cell types. Multiple approaches have been performed to isolate genes differentially expressed in cells containing aberrantly activated MYC proteins leading to the identification of thousands of putative targets. Functional analyses of genes differentially expressed in MYC-transformed cells had revealed that so far more than 40 upregulated or downregulated MYC targets are actively involved in cell transformation or tumorigenesis. However, further systematic and selective approaches are required for determination of the known or yet unidentified targets responsible for processing the oncogenic MYC program. The search for critical targets in MYC-dependent tumor cells is exacerbated by the fact that during tumor development, cancer cells progressively evolve in a multistep process, thereby acquiring their characteristic features in an additive manner. Functional expression cloning, combinatorial gene expression, and appropriate *in vivo* tests could represent adequate tools for dissecting the complex scenario of MYC-specified cell transformation. In this context, the central goal is to identify a minimal set of targets that suffices to phenocopy oncogenic MYC. Recently developed genomic editing tools could be employed to confirm the requirement of crucial transformation-associated targets. Knowledge about essential MYC-regulated genes is beneficial to expedite the development of specific inhibitors to interfere with growth and viability of human tumor cells in which MYC is aberrantly activated. Approaches based on the principle of synthetic lethality using MYC-overexpressing cancer cells and chemical or RNAi libraries have been employed to search for novel anticancer drugs, also leading to the identification of several druggable targets. Targeting oncogenic MYC effector genes instead of MYC may lead to compounds with higher specificities and less side effects. This class of drugs could also display a wider pharmaceutical window because physiological functions of MYC, which are important for normal cell growth, proliferation, and differentiation would be less impaired.

## MYC is an Endpoint of Multiple Signaling Pathways

Cancer cells are featured by deregulated activation and suppression of proto-oncogenes and tumor suppressor genes, respectively. Tumor cells evolve from a multistep process, resulting in sustained proliferation, inactivation of growth suppressors, immortalization, accelerated angiogenesis, metastasis, and resistance to programed cell death. In normal tissues, growth-promoting signals are carefully controlled leading to cellular homeostasis, whereas in cancer cells, these biological signals are deregulated. Signals are transmitted by growth factors, which bind to cell surface receptors containing intracellular tyrosine kinase domains. From here, the signal branches into multiple and complex signal transduction pathways to regulate cell cycle progression, cell growth, survival, and energy metabolism (1). Key players in these processes are encoded by genes, which are normally required to coordinate proper cell metabolism, proliferation, and differentiation. The functions of many of these genes had been elucidated after their identification as transforming principles in oncogenic retroviruses, which carry mutated versions in their genomes (2).

**Figure 1:**
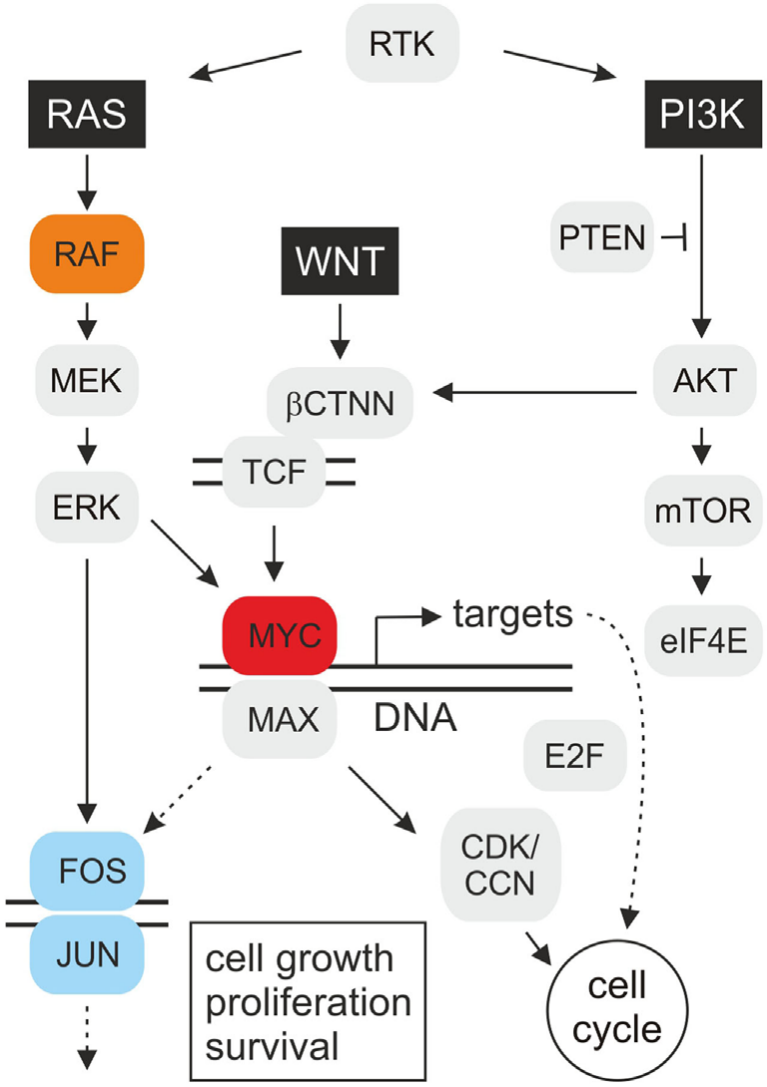
**Schematic depiction of oncogenic MYC signal transduction**. The highly simplified cartoon shows key pathways operating upstream and downstream of the MYC oncoprotein. Mitogenic signal transduction starts with stimulated receptor tyrosine kinases (RTK) transmitting signals *via* guanine nucleotide exchange factors onto the G protein RAS. RAS binds and activates the serine/threonine protein kinase RAF(Mil), which leads to consecutive phosphorylation of the mitogen-activated protein kinase kinase (MAPKK) MEK, the MAP kinase (MAPK) ERK, and of transcription factor complexes, such as MYC/MAX or JUN/FOS (AP-1), regulating the expression of numerous target genes. Based on the observed synergy between MYC and RAF(Mil), distinct MYC targets may enhance RAS/RAF-induced cell transformation *via* a positive feedback loop. On the other hand, MYC could also directly stimulate AP-1 by transcriptional activation of *JUN* or *FOS* encoding genes. The c-*MYC* gene is activated at the transcriptional level by the wingless/int-1 (WNT) signaling pathway, resulting in nuclear translocation of β-catenin (βCTNN) where it binds to T-cell factor (TCF). Several transforming MYC targets are involved in cell cycle regulation (Table 1), which encode *inter alia* E2F transcription factors, cyclins (CCN), and cyclin-dependent kinases (CDK), resulting in accelerated cell proliferation. Besides the extracellular signal-regulated kinase (RAS–ERK) pathway, phosphatidylinositol 3-kinase (PI3K)–mammalian target of rapamycin (mTOR) signaling is a central mechanism to control cell growth, proliferation, and survival in response to extracellular stimuli. The protein kinase AKT phosphorylates many survival factors, and mTOR-mediated signaling modulates ribosome biogenesis and translation of proteins, such as c-MYC and cyclin D, that promote cell growth and proliferation [adapted from Ref. (1, 4, 5)].

Some of the most intensively studied oncogenes encode transcription factors that are functionally located at the end of several signaling cascades, thereby integrating multiple cellular signals. Transcription factors regulate gene expression and similar to cytoplasmic key regulators, the deregulation of many transcription factors is associated with human oncogenesis. Transcription factors bind to the DNA control regions of target genes and activate or suppress their expression, which is important for cell proliferation and differentiation. In case of aberrant gene regulator activities caused by mutations, distinct target genes become abnormally activated or deactivated, which can ultimately lead to oncogenic transformation and malignant cell growth. MYC represents a prototypic transcription factor and a nuclear end point of several signaling pathways (3) (Figure 1). Hence, identification and characterization of transformation-relevant target genes acting downstream of MYC is a prerequisite to understand molecular mechanisms of tumor development in which this oncogenic transcription factor is involved.

## Origin of MYC and Biological Functions

*MYC* has been originally identified as the transforming determinant (v-*myc*) of avian acute leukemia virus MC29 in chicken (myelocytomatosis virus 29) (6). *MYC* was also isolated from the avian leukemia- and carcinoma-inducing MH2 virus, which carries in addition the v-*mil*(*RAF*) allele encoding a serine/threonine protein kinase (7). The presence of two oncogenes significantly increases the oncogenicity of MH2, which is due to cooperative effects between the v-Myc and v-Mil(RAF) proteins (8, 9).

The v-*myc* allele is derived from the cellular c-*myc* proto-oncogene by retroviral transduction (10, 11). C-*MYC* encodes the c-MYC protein, a transcription factor with oncogenic potential representing the central hub of a network controlling the expression of at least 15% of all human genes, and regulating fundamental cellular processes, such as growth, proliferation, differentiation, metabolism, pluripotency, and apoptosis (3, 12). Transcriptional deregulation of human c-*MYC* caused by chromosomal translocation was first observed in Burkitt’s lymphoma (13).

Besides retroviral insertion or transduction of human c-*MYC* leading to the development of lymphomas and carcinomas, amplification of *MYC* alleles has been observed in colon carcinoma, neuroblastoma, and lung cancer leading to the discovery of the N-*MYC* and L-*MYC* paralogs (11). Constitutive activation of MYC is required for oncogenesis and occurs in many human tumor cell lines indicating that deregulated expression of this oncoprotein may contribute to cancer formation. In fact, besides the K-RAS and B-RAF oncoproteins, c-MYC represents a major driver in human tumorigenesis (11, 14). Ectopic expression of c-MYC suffices to induce metastasis in a murine non-small-cell lung cancer (NSCLC) model featuring the most lethal human cancer due to its high metastasis rate. Likewise, in prostate and pancreatic cancer, c-MYC is upregulated upon constitutive stimulation of the RAS and WNT pathways (15–19) (Figure 1). Immortalization and transformation of human epithelial cells occur after overexpressing c-MYC and simultaneously inactivating cyclin-dependent kinase inhibitor 2A (CDKN2A), leading to specific gene expression changes (20, 21). Today, it is known that deregulation of *MYC* genes is a frequent event in animal and human tumorigenesis taking place in more than 50% of all human cancers (3, 22). MYC proteins therefore belong to those crucial master switches in most human cancers, from which many of them are associated with a poor clinical outcome (12, 23).

## Principal Biochemical Functions of MYC

MYC is a bHLHZip protein encompassing protein dimerization domains (helix–loop–helix, leucine zipper) and a DNA contact surface (basic region) that forms heterodimers with the MAX protein and binds typically to specific DNA sequence elements termed E-boxes (5′-CACGTG-3′) (10, 11). MYC and MAX homologs with conserved basic functions were found in primitive metazoans (24, 25) and premetazoans (26), suggesting that principal functions of the MYC master regulator arose very early in the evolution of multicellular animals.

MYC is regulated at transcriptional and translational levels and stabilized by post-translational modifications, such as RAS-dependent phosphorylation (27). In fact, it has been shown that RAS/ERK and PI3K/AKT signaling cascades significantly increase the half-life of MYC, which is normally subjected to rapid ubiquitin-mediated protein degradation (28, 29) (Figure 1). Although MYC is also involved in DNA replication and cell cycle checkpoint processes (30), its major function is transcriptional regulation (11, 12). MYC binds to multiple coactivators representing components of histone acetyltransferase complexes, to ubiquitin ligases, or to other transcription factors, thereby inducing transcriptional activation or repression (10, 11).

## Amplification of Gene Expression by MYC

Previous global analyses, using techniques such as serial analysis of gene expression, DNA microarrays, chromatin immunoprecipitation coupled with high through-put sequencing (ChIP-Seq), promoter scanning, or proteomics, have led to the identification of thousands of genes controlled by the MYC/MAX network, which are involved in fundamental cellular processes, including growth, proliferation, metabolism, differentiation, and apoptosis (31–37). Many of the MYC-activated genes are broadly related to processes of nucleotide synthesis, cell growth, and metabolism, including protein synthesis, ribosomal biogenesis, glycolysis, mitochondrial function, and cell cycle progression (11, 12, 38). In addition, several cell cycle-related genes whose protein products initiate DNA replication are transcriptional MYC targets, which could explain why deregulated DNA synthesis, chromosomal abnormalities, and genomic instability frequently occurs in human tumor cells containing activated MYC (39).

Deregulated MYC target genes have been identified in numerous human tumors (11, 40), but so far it has been difficult to ascribe the oncogenic properties of MYC to a defined set of target genes. In fact, results from recent studies indicate that MYC acts as a general amplifier of gene expression (41–43). According to this theory, the promoters of all actively transcribed genes are occupied and activated by c-MYC in tumor cells expressing high levels of this transcription factor, leading to non-linear amplification of existing transcriptional activities (41, 42, 44–46). The observed differential expression of multiple genes in cells containing aberrantly activated MYC is therefore due to individually enhanced gene expression occurring at varying levels. The amplifier model also explains how ectopic c-MYC increases the efficiencies of other transcription factor programs (46), e.g., during generation of pluripotent stem cells from fibroblasts. This re-programing of cells is achieved by overexpressing the transcription factors OCT4, SOX2, and KLF4 (47). On the other hand, gene repression in cells transformed by MYC is caused by MYC interaction with specific transcription factors or indirectly by increasing the expression of repressive transcriptional and chromatin components.

## MYC Targets with Oncogenic or Transformation-Suppressive Activities

The conversion of a normal into a tumorigenic cell could be caused by the products of multiple transformation-associated MYC target genes, from which more than 40 have been identified so far (Table 1). Some of these genes exhibit transforming activity upon ectopic expression, suggesting that they contribute to MYC-induced oncogenesis. Furthermore, there is evidence that MYC enhances the effects of other oncogenic gene regulators, such as E2F (48) or AP-1 (9) (Figure 1). In addition to the implication of MYC/MAX heterodimers in transcriptional activation, MYC has been also associated with transcriptional repression, thereby in many cases not binding directly to E-boxes but instead involving other transcription factors such as MIZ-1 or SP1 (3, 12, 46, 49, 50). Most of the genes repressed by MYC are involved in cell cycle arrest, cell adhesion, and cell-to-cell communication (11).

**Table 1:** **Activated and suppressed MYC target genes associated with cell transformation**.

Gene	Activated (+)/suppressed (−)	Protein product	Function	Transformation association	Reference
*AP4*	+	AP4	Gene regulator	Required for MYC-induced cell cycle progression	(51)
*BMP7*	+	BMP7	Bone morphogenetic protein	Silencing blocks medulloblastoma cell proliferation	(52)
*CCNB1*	+	Cyclin B1	CDK regulatory subunit	Induces tetraploidy upon overexpression	(53)
*CCND2*	+	Cyclin D2	CDK regulatory subunit	Absence inhibits MYC-induced colony formation	(54)
*CCNE1*	+	Cyclin E1	CDK regulatory subunit	Associated with neuroblastoma progression	(55)
*CDC25A*	+	CDC25	Cell cycle phosphatase	Induction of apoptosis in growth factor-depleted cells	(56)
*CDK4*	+	CDK4	Cyclin-dependent kinase	Absence inhibits *MYC*-induced tumor development	(57)
*CDT1*	+	CDT1	Chromatin licensing factor	Colony formation in fibroblasts	(58)
*E2F1*	+	E2F1	Cell cycle regulator	Inhibition of MYC-induced apoptosis	(48)
*GATA-4*	+	GATA-4	Gene regulator	Knock-down inhibits colony formation	(18)
*HMG-I/Y*	+	HMG-I/Y	Chromatin-binding protein	Tumor generation in nude mice	(59)
*HSP90A*	+	HSP90	Heat shock protein	Contributes to MYC-induced transformation	(60)
*JAG2*	+	Jagged2	Notch receptor ligand	Ectopic expression increases tumorigenesis	(61)
*JPO1*	+	JPO1/CDCA7	Nuclear protein	Ectopic expression increases lymphoid maligancy	(62)
*LDH-A*	+	Lactate dehydrogenase	Enzyme in anaerobic glycolysis	Anchorage-independent growth in rat fibroblasts	(63)
*MCL1*	+	Mcl-1	Myeloid cell leukemia protein	Abrogation of MYC-driven lymphoma development	(64)
*MMTN*	+	Mimitin	Mitochondrial protein	Knock-down leads to tumor cell growth arrest	(65)
*MTA1*	+	MTA1	NURD complex component	Knock-down inhibits MYC-induced colony formation	(66)
*MT-MC1*	+	MT-MC1	Nuclear protein	Tumorigenic activity	(67)
*NPM*	+	Nucleophosmin	Nucleolar protein	Enhances *MYC*/*RAS* cotransformation in MEF	(68)
*ODC*	+	Ornithine decarboxylase	Enzyme for polyamine synthesis	Knock-out prevents MYC-induced lymphomagenesis	(69)
*OPN*	+	Osteopontin	Extracellular signaling protein	Colony formation in primary fibroblasts	(9)
*PIN1*	+	PIN1	Peptidyl-prolyl isomerase	Genetic ablation reduces MYC-induced lymphomagenesis	(70)
*PMTA*	+	Prothymosin-α	Chromatin remodeling factor	Induction of anchorage-independent growth	(71)
*PRDX3*	+	Peroxiredoxin	Mitochondrial protein	Colony formation in soft agar	(72)
*PRMT5*	+	Arg methyl transferase	Key enzyme in snRNP assembly	Knock-out in lymphoma cells reduces tumorigenesis	(73)
*RCL*	+	RCL	Nuclear protein	Colony formation in rat fibroblasts	(63)
*TFRC1*	+	TFRC1	Transferrin receptor 1	Enhancement of MYC-mediated tumor formation	(74)
*Tmp*	+	Tmp	Tumor-associated glycoprotein	Tumor formation in nude mice	(75)
*WS5*	+	WS5/Pmel17	Transmembrane glycoprotein	Colony formation in primary avian fibroblasts	(76)
*p32*	+	C1QBP	Complement component	Inhibition of tumor cell growth upon knock-down	(77)
*lnc H19*	+	n.a.	Long non-coding RNA	Knock-down decreases cancer cell clonogenicity	(78)
*BASP1*	−	Brain acid-soluble protein	Signaling protein	Inhibition of focus and colony formation	(50)
*FER-H*	−	Ferritin H	Iron storage protein	Downregulation required for oncogenesis	(79)
*NDRG1*	−	N-*myc* downregulated gene	Hydrolase	Metastasis suppressor	(80)
*PRDM11*	−	PR-domain protein	Transcriptional regulator	Knock accelerates MYC-induced lymphomagenesis	(81)
*Onzin*	−	Onzin	Cysteine-rich protein	Oncogenesis upon overexpression^a^	(82)
*THBS1*	−	Thrombospondin	Antiangiogenic factor	Overexpression reduces tumorigenesis in xenografts	(83)
*TXNIP*	−	Thioredoxin-interacting protein	Negative regulator of glycolysis	Expression reduces cell proliferation	(84)
*MycLo4-6*	−	n.a.	Myc-repressed lncRNAs	Prohibits MYC-enhanced cell proliferation	(85)

*n.a., not applicable*.

*^a^Disproportional behaviour*.

Besides regulating the expression of protein-encoding genes, MYC also controls the expression of distinct long non-coding ribonucleic acids (lncRNA) (78, 85–87) and of multiple small non-coding regulatory microRNAs (miRNA) (3, 88–91). Some of the miRNAs have oncogenic properties such as the miR-17–92 cluster (oncomir-1) or have tumor suppressor functions (16, 85, 88, 92) (Table 1). miRNAs inhibit protein translation or lead to degradation of their target messenger RNAs (mRNA) and have been implicated in cancer by inactivating distinct mRNAs encoding oncogenes or tumor suppressors (93).

## Approaches to Identify Critical Targets Executing MYC-Induced Cell Transformation

Transformation-associated targets of MYC either display intrinsic transforming activities or inhibit oncogenesis depending whether they are activated or repressed (Table 1). Assuming that MYC transformation is mainly based on transcriptional deregulation, only the combined effects of multiple activated or suppressed targets may suffice to induce a “MYC-like” transformed phenotype. However, just systematically analyzing known transformation-relevant MYC targets is not constructive because the list in Table 1 is not exhaustive, and even more important, many of these targets have been isolated from different cellular systems under *in vitro* cell culture conditions. This may not reflect the real situation *in vivo* in which oxygen, nutrients, or growth factors are limited. Furthermore, the hypothesis in earlier reductionists’ approaches assuming that a tumor consists of a homogenous collection of cancer cells and its biology is accessible by elucidating all cell autonomous properties and is not valid any more. In human carcinogenesis, diverse cell types from cancer stem cells give rise to intra-tumor heterogeneity, thus further increasing the genetic complexity and representing a major cause of cancer recurrence (1, 94, 95). Cancer cells progressively evolve from normal cells in a multistep process, thereby acquiring distinct characteristic features in an additive manner. Thus, a succession of clonal expansions occurs also involving epigenetic mechanisms such as methylation or histone modification. In particular, the transition to invasion and metastasis encompasses several discrete steps. With regard to this complex scenario, certain *in vitro* environmental pertubations have to be reconsidered and better adapted to the *in vivo* situation, for instance, by using isogenic cell lines, which differ only in single allelic mutations (95, 96). More unbiased approaches based on oncogenic functions are required to identify a putative magic target gene set which suffices to phenocopy MYC transformation, supposed that such Holy Grail exists at all. The following approaches are suggested to dissect the complexity of the oncogenic MYC transcriptional program:

### Functional Expression Cloning

Isolation of novel coding and non-coding MYC targets with strong oncogenic activities could be done by cDNA expression cloning using MYC-dependent tumor cells as a source for RNA isolation. The application of retroviral cDNA expression libraries has already successfully led to the isolation of transforming genes from human tumor cells (97–100). Thereby, the selection for distinct genes is based exclusively on function, in this case the capacity to transform cells. Appropriate gene-transfer tools are retroviral vectors, allowing the efficient introduction of complex cDNA libraries (99, 101) and appropriate screening procedures.

### Combinatorial Gene Expression

Due to the pleiotropic MYC effect leading to the development of multiple different tumor forms, one could postulate that simultaneous perturbation of multiple targets suffices to convert a normal cell into a cancer cell displaying a MYC-transformed phenotype. Due to the capacity of MYC to enhance existing transcriptional programs (see above), the identification of transcription factors, which are involved in executing the oncogenic MYC program, should be straightforward. Critical MYC targets can then be overexpressed and inactivated depending on whether they are activated and suppressed in MYC-transformed cells, respectively. To simultaneously overexpress multiple genes or interfering RNAs in single cells, several established techniques exist. They are based on different principles such as co-transfection of multiple plasmids, usage of bicistronic vectors containing an internal ribosomal-binding site, infection with retroviruses containing different envelope subtypes, or self-processing peptides (47, 102).

### Analysis of Targets by Permanent Gene Inactivation

The functionality of critical target genes can be tested by genomic inactivation and the usage of appropriate *in vivo* tumor model systems. To analyze if expression of a distinct target is required for maintenance of cell transformation, its inactivation should be performed in MYC-dependent tumor cells. Otherwise, to test if a target is required for the initiation of MYC-induced cell transformation, the relevant gene has to be disrupted in normal cells prior to *MYC* transduction. An appropriate tool for genomic inactivation is the recently developed clustered regularly interspaced short palindromic repeats (CRISPR) system (103, 104). Precise genome editing is achieved by creating specific double-stranded breaks, which allow the generation of homozygous knock-out or knock-in genotypes. Specific MYC target gene inactivation could lead to inhibition of the tumorigenic phenotype, cell cycle arrest, or apoptosis. Suitable *in vivo* techniques to quantify gene inactivation effects on tumor growth and angiogenesis are, e.g., the generation of mouse xenoplants, and the chicken chorioallanthoic membrane assay. Inhibition of tumorigenesis caused by inactivation of MYC distinct targets would indicate essential functions of the tested genes.

## MYC Targets as Templates for Inhibitor Design

Because of its pivotal role in cancer, MYC has become an obvious target in the treatment of human cancer cells. Several approaches to interfere with *MYC* gene transcription, MYC protein function, or with the functions of distinct targets have been pursued to inhibit MYC-dependent pathogenesis.

Intracellular signal transduction pathways regulating MYC expression and protein stability have been targeted by using chemical inhibitors, which are in the trial phase or already applied in the clinic. Thereby, key proteins of the two main signaling cascades responsible for cell survival, differentiation, proliferation, metabolism, and motility were inhibited: the RAS–extracellular signal-regulated kinase (ERK) and the phosphatidylinositol 3-kinase (PI3K) pathways (4, 105, 106) (Figure 1).

Direct inhibition of MYC functions has been achieved by using different strategies. c-MYC transcription has been targeted by inhibiting the chromatin acetyl-lysine recognition domain (bromodomain) of a MYC-specific coactivator. This led to suppression of c-MYC transcription followed by genome-wide downregulation of MYC-dependent target genes (107). C-*MYC* transcription has been blocked by the miRNA miR-494 leading to inhibition of proliferation, invasion, and chemoresistance in pancreatic cancer (108). Furthermore, a dominant negative mutant of the MYC dimerization domain termed Omomyc is effective against glioma thereby inhibiting cell proliferation and increasing apoptosis (109). Perturbation of MYC/MAX interaction by synthetic α-helix mimetics or by the homeobox protein Hhex led to impaired DNA binding suppressed transcriptional activation and inhibition of cell growth and tumorigenesis (110, 111). Efficient interference with MYC functions has been also achieved by using novel pyridine inhibitors leading to specific inhibition of MYC/MAX dimerization, transcriptional regulation, and oncogenesis (112, 113). These novel compounds reveal a unique inhibitory potential even at nanomolar concentrations combined with the specific inhibition of MYC-driven tumor growth *in vivo* (112).

However, under normal physiological conditions MYC is required for many cell physiological processes and for homeostasis. A complete block of the MYC protein by binding to efficient inhibitors may result into undesired side effects or into drug resistance after prolonged application. Approaches based on the principle of synthetic lethality using MYC-overexpressing cancer cells have lead to the identification of targets, which may be susceptible towards appropriate drugs. Synthetic lethality is defined by cell death induced by mutation or inhibition of two different genes, whereas the dysfunction of one gene has no effect on cell viability. This principle can be exploited to screen for anticancer drugs by mimicking the effect of the second genetic mutation using chemical or inhibiting-RNA libraries (114). For instance, pharmacological inhibition of the eukaryotic translation factor eIF4F is synthetic lethal in an *E*μ-*MYC* lymphoma model (115). Likewise, selective death of MYC-dependent human breast cancer cells was achieved by siRNA-mediated inhibition of cyclin-dependent kinase 1 (CDK1) (116). Another example is the identification of a DNA repair protein kinase (PRKDC) as a synthetic lethal target in MYC-overexpressing lung cancer cells, which was identified in RNAi library screen (117). Therefore, attacking oncogenic MYC effectors may increase the specificity of MYC-dependent tumor treatment and enlarge the arsenal of available drugs.

## Author Contributions

The author confirms being the sole contributor of this work and approved it for publication.

## Conflict of Interest Statement

The author declares that the research was conducted in the absence of any commercial or financial relationships that could be construed as a potential conflict of interest.
